# Analytical Study of Hybrid Techniques for Image Encryption and Decryption

**DOI:** 10.3390/s20185162

**Published:** 2020-09-10

**Authors:** Chiranji Lal Chowdhary, Pushpam Virenbhai Patel, Krupal Jaysukhbhai Kathrotia, Muhammad Attique, Kumaresan Perumal, Muhammad Fazal Ijaz

**Affiliations:** 1 School of Information Technology and Engineering, Vellore Institute of Technology, Vellore, Tamil Nadu 632014, India; chiranji.lal@vit.ac.in (C.L.C.); pkumaresan@vit.ac.in (K.P.); 2 School of Computer Science and Engineering, Vellore Institute of Technology, Vellore, Tamil Nadu 632014, India; pushpam.patel2017@vitstudent.ac.in (P.V.P.); krupal.kathrotia2017@vitstudent.ac.in (K.J.K.); 3 Department of Software, Sejong University, Seoul 05006, Korea; attique@sejong.ac.kr; 4 Department of Intelligent Mechatronics Engineering, Sejong University, Seoul 05006, Korea

**Keywords:** cryptography algorithm, elliptic curve cryptography, hill cipher, advanced encryption standard, double playfair cipher, encryption and decryption, peak signal to noise ratio, Number of Pixels Change Rate (NPCR), Unified Average Changing Intensity (UACI), lossy compression

## Abstract

The majority of imaging techniques use symmetric and asymmetric cryptography algorithms to encrypt digital media. Most of the research works contributed in the literature focus primarily on the Advanced Encryption Standard (AES) algorithm for encryption and decryption. This paper propose an analysis for performing image encryption and decryption by hybridization of Elliptic Curve Cryptography (ECC) with Hill Cipher (HC), ECC with Advanced Encryption Standard (AES) and ElGamal with Double Playfair Cipher (DPC). This analysis is based on the following parameters: (i) Encryption and decryption time, (ii) entropy of encrypted image, (iii) loss in intensity of the decrypted image, (iv) Peak Signal to Noise Ratio (PSNR), (v) Number of Pixels Change Rate (NPCR), and (vi) Unified Average Changing Intensity (UACI). The hybrid process involves the speed and ease of implementation from symmetric algorithms, as well as improved security from asymmetric algorithms. ECC and ElGamal cryptosystems provide asymmetric key cryptography, while HC, AES, and DPC are symmetric key algorithms. ECC with AES are perfect for remote or private communications with smaller image sizes based on the amount of time needed for encryption and decryption. The metric measurement with test cases finds that ECC and HC have a good overall solution for image encryption.

## 1. Introduction

An image provides a lot of information. Almost one-third of our cortical brain region is dedicated to the visual processing of the perceived information. Images are a significant source of information. Images have various applications in a variety of fields such as storing patient medical information, capturing aerial images by satellite imagery, capturing interplanetary motion images by telescopes, storing an individual’s identity in the form of fingerprints, or iris images, etc. [1]. Digital communication generates millions of digital data in the form of digital images. Cryptography is an efficient way to safeguard sensitive information. Cryptography is a method of storing and transmitting data in a form intended for reading and processing the information. The advancement of encryption and decryption leads to an infinite future. Security analysis depicts the schematic encryption the scheme can endure numerous crypt analytical attacks. Reliability and protection of information are equally critical to obtain respect from the recipient for the information obtained [2].

Encryption of images plays a key role if the images are to be kept private and transmitted securely. The encryption task involves distorting the pixel intensity of the image input to create a cipher image that is completely different from the image input. Using the secret keys, the receiver decrypts the images and returns the original image. There are various private keys used by the sender and receiver in asymmetric key cryptography which are further used to generate the shared secret key. On the other hand, symmetric-key cryptography involves encryption and decryption with a single key that the sender and receiver are secretly known to have [3].

Most common processes involve symmetric approaches such as AES, DES, Hill cipher, etc. to protect the information stored in the images [4]. While they are easy to implement and fast to process, the amount of security given to the image is lacking for these methods. This is overcome by incorporating asymmetric techniques such as RSA, ECC, ElGamal, etc. that provide more security but a trade-off in ease of implementation and feasibility of computations [5,6]. Saied et al. [7] used an artificial neural network (ANN) algorithm in encryption/decryption based on specific characteristic features. However, these processes become more difficult to deploy with time complexity when communicating several encryptions and decrypting parameters through an unsecured channel. Elliptic curve and ElGamal cryptosystems include asymmetric key cryptography, while Hill Cipher, AES, and Double Playfair Cipher are symmetric key algorithms [8,9]. Asymmetric key cryptography is highly secure but requires significant computational complexities that are further eased using asymmetric key cryptography algorithms in addition to asymmetric key cryptography [10,11].

Digital image encryption and decryption provides full data protection for the digital image pixels in our proposed research work. To transfer images quickly, we use a hybrid encryption technique. It provides safety to different devices such as mobile phones, tablets, etc. The system ensures the integrity of the data that does not change the information transferred. Since data encryption applies to both rest and transit information, it provides consistent security that could result in peace of mind for the people who handle the information. Compression results in reduced disk consumption and increased write speed, reading speed, and fast file transfer.

The main contributions of this paper are:

- A new image encryption approach using symmetric hybrid algorithms ECC with Hill Cipher, ECC with AES, and ElGamal with Double Playfair Cipher.
- The hybrid decryption algorithms using random permutation and dynamic keys.
- Our proposed hybridization of algorithm ensure the ease of implementation with increase in speed from symmetric algorithms. This also improved the security from asymmetric algorithms.
- The efficiency of the proposed algorithm is seen in several tests and comparisons performed.
- The results show that the proposed algorithm is effective to outperform some key cryptographic algorithms.

The remainder of the paper is organized as follows. Section 2 presents a literature survey. The background study for proposed hybrid algorithms is covered in Section 3. The proposed image encryption and decryption implementation are given in Section 4. Section 5 presented the performance of the proposed approach. The succeeding Section 6 describes the evaluation and result from the analysis of the proposed algorithm. Section 7 shows result discussion, followed by a comparative analysis of proposed hybrid algorithms with an existing algorithm. Finally, the conclusion is presented in Section 8.

## 2. Literature Review

This section deals with related work in the field of image encryption and decryption. Security becomes a significant problem in today’s environment, for both storing and transmitting multimedia data. Therefore, data must be protected from unauthorized access and the attacker must thwart the attack during transmission. Images have a major role to play in multimedia data. This represents more information when compared with text details through visualization [12,13]. The application of images in healthcare, education, transportation and law enforcement, military communication, medical sciences, etc. requires high image data security only between end-users. Encryption is the mechanism that renders image unreadable or makes it easy to evaluate [14,15].

Numerous works have been performed to make ECC encryption possible after each pixel has been mapped to a predefined elliptic curve [16,17,18]. The algorithm is used to perform faster, but complicated in pre-computation calculations to find each point of the elliptical curve for a large value of the primary number used to generate the finite field. It also includes communication of the wide mapping table through the unreliable channels for the decryption process. Several research studies have focused mainly on the use of AES as an encryption and decryption algorithm [19]. While the algorithm claims to perform better than other methods, it did not include concrete results for the same through a variety of security and encryption quality metrics, such as Entropy, NPCR, UACI, etc. [20].

The various approaches involve AES with visual cryptography which offers good results by encrypting the image using AES and the original key using visual cryptography by converting it to an image. The algorithm is still susceptible to an attack on the shared image created for the key [21,22]. Hashim et al. [23] used ElGamal encryption as an asymmetric encryption algorithm and have been tested using MATLAB. The work concluded that it took an increasing amount of time for computation using a large prime number as the encryption parameter.

Bhowmick et al. [24] worked on the security of the text encryption provided by Double Playfair Cipher by using 6 × 6 key matrices over the regular 5 × 5 key matrices. However, the algorithm failed due to data loss over certain characters, such as spaces and special symbols. An updated 5 × 5 playfair cipher version is presented which enables the user to encrypt and decrypt messages for any square matrix. The fair play cipher, with the unique encoding instructions, is introduced as the first digraph cipher. Hardi et al. [25] combined the use of the ElGamal cryptosystem and the Double Playfair cipher to protect text data using standard keys for the symmetrical method. While the algorithm appears to be operating on digital media, it fails to evaluate security measures and metrics. Image file encryption performed using hybrid cryptography. ElGamal algorithm used to perform asymmetric encryption and Double Playfair for symmetric encryption. The result has been proved that these algorithms are capable of encrypting an image file with an appropriate runtime and encrypted file size while maintaining the security level.

Hamad et al. [26] worked to increase image encryption protection utilizing standard Playfair cipher using a modified key of size 16 by 16 on the 8-bit pixel range. By carrying out an XOR operation using a random mask, the effects are further enhanced. The additional security of the algorithm through the XOR function fails if the intruder eavesdrops the mask. An algorithm incorporating XOR encryption with a rotational process was designed to effectively encrypt images. Arab et al. [27] proposed the Advanced Encryption Standard (AES) and Visual Cryptographic techniques for images. Secure image encryption algorithm used for both AES and Visual Cryptographic techniques to protect the image. The image is encrypted using AES and an encoding schema has been proposed to convert the key into shares based on Visual Secret Sharing.

Astya et al. [28] introduced the Cryptography of the Elliptic Curve for images. The proposed work is designed to provide secure authentication to combine image encryption with Elliptic Curve Cryptography. The matrix operations are carried out on the original image matrix, and the transformed image is further encrypted using a key sequence generated from the elliptic curve. This is a highly secure technique and difficult to get the original data without the key. The system requires high computation which makes it slower. Dawahdeh et al. [29] have adopted the encryption technique incorporating the Elliptic Curve Cryptosystem with Hill Cipher. The researchers selected ECC asymmetric encryption and Hill Cipher for symmetric encryption. The proposed algorithms are capable of encrypting an image file with security measures. More secure as a hybrid approach is used, and faster in computing. A new self-invertible key matrix technique was proposed. It uses single matrix to encrypt the pixels within the image but it takes longer.

Anwar et al. [30] suggested Elliptic Cryptosystem based curve, which is an efficient public-key cryptosystem and more suitable for limited environments. The efficiency of the elliptical curve cryptosystem depends heavily on the operation called point multiplication. This is a highly secure technique and very difficult to get the original data without a key. More computation is required, making the system slower. Nagaraj et al. [31] considered a challenging task due to latency, key size, and security issues with the appropriate algorithms for specific application. Cryptographic algorithms face various types of attacks, such as brute force attacks, a man in the middle attack, loop attacks, etc. which remain as threads. The researcher provides suggestions about the algorithms to be used according to needs. The concept is either symmetric or asymmetric but not a combination that limits their functionality.

The work in [32] used a 5D hyperchaotic system with 2 positive Les (Lyapunov exponent), pixel-level dynamic filtering, DNA computing, and 3D Latin cubes for image encryption. Interestingly, the work in [33] presented two cryptosystems approaches as partial encryption and S-box. The approach to encryption shows high results in simulations. Another mechanism in [34] mentioned a dynamic state variables selection to dynamically assign chaotic variables for pixel encryption. The authors in [35] have introduced a cosine-transform-based chaotic system (CTBCS). The encryption method requires higher-efficiency scrambling to distinguish neighboring pixels and uses random order substitution to distribute a small shift to all pixels of the cipher-image in the plain-image.

## 3. Background Study for Proposed Hybrid Algorithms

In our research work, there are three different hybrid methods for performing image encryption and decryption. The key generation of ECC and background process describes the hybrid algorithms mentioned below.

- ECC with Hill Cipher,
- ECC with AES,
- ElGamal with Double Playfair Cipher.

### 3.1. Key Generation of ECC

The overview of an Elliptical Curve Cryptography is specified. Elliptical curves are used across a finite prime field. Mathematically the curve is defined as:

(1) $$\left(F^{p})=\{a,b,p,G\right\}$$

(2) $$y^{2}\equiv x^{3}+ax+b\left(mod\;p\right)\;\mathrm{and}\;4a^{3}+27b^{2}≢0\left(mod\;p\right)$$

where $F_{p}$ is the finite field over a prime number p with generator *G*. *a*, *b* are curve parameters.

The generator of the curve *G* is the point whose point multiplication with different scalars produces every point on the curve. Further, we define the order of elliptic curve *n* as the smallest integer whose scalar point multiplication with generator *G* gives us the point at infinity *O* for the curve, i.e., *nG* = *O*. For the proposed algorithms we need to generate the keys for elliptic curve cryptography. The key generation algorithm is given below in Algorithm 1.
**Algorithm 1:** Key Generation  Receiver establishes Elliptic curve parameters: (Fp)={a,b,p,G};  Choose a private key *nB* in the range: {1,p−1};  Find the public key: *PB* = *nBG* point multiplication over the curve;  Publish the public key *PB* and the curve parameters (Fp)={a,b,p,G};  Sender gets the public key of receiver PB along with associated curve parameters;  Sender chooses a private key nA in the range: {1,p−1};  Sender computes his public key as PA=nAG;  The shared secret key (SSK) as computed by the sender will be:  (SSK)=nAPB=nAnBG=(x,y);

The key generation is added in our proposed algorithm that uses ECC as the asymmetric approach.

### 3.2. ECC with Hill Cipher Algorithm for Image Encryption and Decryption

There is hybridization of image encryption and decryption algorithms of ECC with Hill Cipher. The matrix generated for HC is a self-invertible matrix is $K_{m}=K-1$. The size of self-invertible matrix used is 2×2. With 4×4 size self-invertible matrix performs the encryption and decryption process comparatively faster as well as produces more distortion in the encrypted image by using maximum number original image pixels to generate the corresponding cipher pixels. The matrix is extracted from the shared secret key, the matrix need not be sent with the encrypted image. The algorithms in the work focus on encryption of grayscale images and given in Algorithm 2.

**Figure 1:**
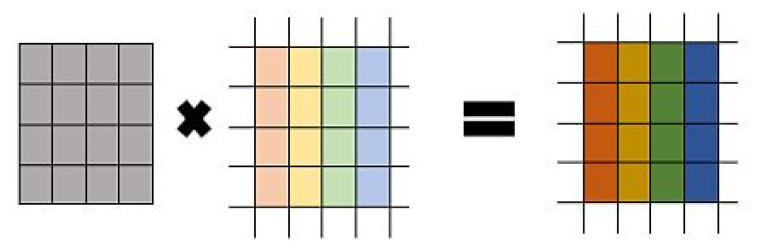
Elliptic Curve Cryptography (ECC) with hill cipher.

ECC with Hill Cipher methodology have gained immense momentum as key picture and shared by both sender and receiver. The encrypted image is formed when the gray scale image is mixed to the Hill Cipher algorithm. The inverse of image K-1 is obtained by the receiver. The resulting encrypted image at the receiver is passed to the Hill Cipher in order to obtain the original image. This mechanism is illustrated in Figure 1. **Algorithm 2:** ECC with Hill Cipher  **Input**: The image of size 256 ×256 to be encrypted or decrypted.  Elliptic curve parameters: (Fp)={a,b,p,G}.  **Output**: The corresponding cipher image or original image of size 256 × 256.  1.  Key Generation for ECC (The ECC keys are generated as shown in Section 3.1, Algorithm 1;  2.  Computing the self-invertible matrix    (a)  Compute: $K_{1}=xG=\left(k_{11},k_{12}\right)$ and $K_{2}=yG=\left(k_{21}+k_{22}\right)$;    (b)  Compute: $K_{12}=I_{2}-k_{11};K_{21}=I_{2}+k_{11}$; and $k_{11}+k_{22}=0$;    (c)  The 4×4 self-invertible matrix is derived as:
$$K_{m}=\left[\begin{array}{cc} k_{11} & k_{12}\\ k_{k21} & k_{22} \end{array}\right]$$       where, $K_{11}=\left[\begin{array}{cc} k_{11} & k_{12}\\ k_{21} & k_{22} \end{array}\right]$  3.  Encryption process:    (a)  Read the image to be encrypted and collect the image pixels separately for the       channels *R*, *G*, and *B*.    (b) Group every channel of pixels into 4 × 4 matrices and perform matrix multiplication       with computed self-invertible matrix.    (c) The encryption is done using the subsequent formula:        $\begin{array}{c} C_{i}=K_{m}\cdot P_{i}=\left[\begin{array}{cccc} k_{11} & k_{12} & k_{13} & k_{14}\\ k_{21} & k_{22} & k_{23} & k_{24}\\ k_{31} & k_{32} & k_{33} & k_{34}\\ k_{41} & k_{42} & k_{43} & k_{44} \end{array}\right]\left[\begin{array}{cccc} p_{11} & p_{12} & p_{13} & p_{14}\\ p_{21} & p_{22} & p_{23} & p_{24}\\ p_{31} & p_{32} & p_{33} & p_{34}\\ p_{41} & p_{42} & p_{43} & p_{44} \end{array}\right]=\left[\begin{array}{cccc} c_{11} & c_{12} & c_{13} & c_{14}\\ c_{21} & c_{22} & c_{23} & c_{24}\\ c_{31} & c_{32} & c_{33} & c_{34}\\ c_{41} & c_{42} & c_{43} & c_{44} \end{array}\right] \end{array}$
       where, $K_{m}$ is the self-invertible matrix and $P_{i}$ is the current input image block to be encrypted;    (d) Allocate the cipher pixels exactly to the same position as of the corresponding input       image pixels. A cipher image is formed of size identical to the size of input image.    (e) Send the cipher image and ECC public key to the receiver. The encryption process       can be visualized as in Figure 1.  4.  Decryption process    (a) Compute the shared secret key from the public key of sender as:       SSK=nBPA=nBnAG=nAnBG=(x,y)    (b) Compute the self-invertible matrix exactly as described above using the shared       secret key.    (c) Group the cipher pixels into 4×4 matrices and perform matrix multiplication with       self-invertible matrix from Step 2.    (d) Allocate the plain text pixels to the same position as of the corresponding cipher       pixels. The original image is formed back and retrieved as without any intensity loss.

### 3.3. ECC with AES for Image Encryption and Decryption

Hybrid algorithms Elliptic Curve Cryptography (ECC) with AES is performed for image encryption and decryption (Algorithm 3). AES key and initialization vector (IV) must be of equal length and it should be a multiple of 16 or 24 or 32 bytes respectively for AES-128, AES-192 or AES-256 encryption/decryption. The AES encryption and decryption is performed using the Cipher Feedback (CFB) mode. If the Electronic Code Book (ECB) mode is used then the initialization vector (IV) is not required. The AES encrypted bytes are converted to large integers to save the number of operations by encrypting 2 × *groupsize* number of bytes in one ECC operation. Base 256 representations is chosen as it would have values 0 to 255 in it and the algorithm is working on an 8-bit image whose pixel intensities also range from 0 to 255. While performing ECC encryption, we make use of point addition formula to encrypt any point on the XY-plane. The advantage of such an operation saves from creating a mapping table which becomes computationally impossible if a very large prime number is used for generating the finite field and sharing it between the users. On performing decryption simply reflect the SSK co-ordinates for the x-axis and taking modulus *p*. The reflected point is then used with the point addition formula for performing the inverse operation used an encryption method.
**Algorithm 3:** ECC with AES  **Input**: The image of size 256 × 256 to be encrypted or decrypted.  Elliptic curve parameters: (Fp)={a,b,p,G}.  AES symmetric key and initialization vector (IV).  **Output**: The corresponding cipher image or original image of size 256 × 256.  1.  Key Generation for ECC    (The ECC keys are generated as shown in Section 3.1);  2.  Key Generation for AES    (a) Sender randomly generates 16 bytes long AES symmetric key and the       Initialization Vector;    (b) These parameters must be securely transmitted to receiver;  3.  AES Encryption    (a) Read the image to be encrypted and collect the image pixels separately for the       channels *R*, *G*, and *B*.    (b) Convert the pixels in each channel to bytes using the function (*PL*) where *PL*       denotes the list of pixels. The function returns an immutable array of bytes.    (c) Perform AES encryption for each of the channel bytes. This generates bytes in the       encrypted form.  4.  ECC Encryption    (a) Choose a base of representing the numbers. We have chosen 256 to represent the       range of 8-bit pixel values;    (b) Represent the prime number *p* in base 256 as a list of integers from 0 to 255.       Measure the length of this representation as *L*. Initialize group size as L−1    (c) Group the cipher bytes from each channel separately with group size initialized       as above. For each of the channels, convert each group of cipher bytes to big       integers treating base of representation as 256. If numbers of big integers are odd,       append with a random value (possibly less than 256);    (d) Pair up the big integers to represent it as a point on the *XY*-plane. If the       *X* co-ordinate of the pair and that of the shared secret key are identical, then we       apply point doubling formula over the pair co-ordinates. Otherwise apply point       addition formula with *SSK* co-ordinates.    (e) From Step 4, we get a point on *XY* plane not at all necessary to be on the       curve chosen. Represent its co-ordinates in base 256 digits. Each of these.       representations must be of size equal to L=groupsize+1. Append 0 zeros       otherwise to make the required length.    (f) The base 256 representations of cipher points are treated as image pixels and cipher       image is constructed correspondingly. The width of cipher image is kept same as       width of input image and height is computed based on the total number of cipher       pixels collected. We observe that size of cipher image is generally more than the       input image.    (g) Send the cipher image, original image size and ECC public key to receiver.  5.  ECC Decryption    (a) Compute the shared secret key from the public key of sender as:       *SSK* = *nBPA* = *nBnAG* + *nAnBG* = (*x*, *y*);    (b) Represent the prime number *p* in base 256 as a list of integers from 0 to 255.       Measure he length of this representation as *L*. Initialize group size as:       groupsize=L−1    (c) Collect the cipher pixels for each channel and group them with each group size       equal to *groupsize* + 1;    (d) Convert each group in respective channels to corresponding big integers taking       base to be 256. Pair up the big integers treating them as points on the *XY*-plane.    (e) Perform reflection of SSK point with respect *x*-axis: (x,−ymodp);    (f) Perform point doubling with reflected SSK co-ordinates if the *x* co-ordinates are       same or perform point addition formula otherwise. The point obtained for each       pair lies on *XY*-plane but need not be a point the elliptic curve    (g) Represent each of the points obtained in base 256 with each representation of       length equal to *groupsize*. Append zeros if required.    (h) Obtained values are bytes obtained from AES encryption.  6.  AES Decryption    (a) Obtain the AES key and IV from secure communication.    (b) Perform AES decryption for the bytes obtained from above for each of the respective       image channels.    (c) Represent the bytes as the plain text image of size as mentioned by the sender.

### 3.4. ElGamal with Double Playfair Cipher for Image Encryption and Decryption

The background work carried out for the ElGamal with Double Playfair Cipher for Image Encryption and Decryption (Algorithm 4). The algorithm uses 2 key matrices for Double Playfair Cipher but the size of these key matrices used is 16 × 16 instead of the traditional 5 × 5 keys used in text encryption. The matrix sizes correspond to the number of distinct pixel values represented by an 8-bit image which is 0 to 255 or 256 distinct values. The use of key maps increases the speed of the algorithm to a great extent by saving the time to traverse the entire key matrices twice in the encryption or decryption process of a single pixel. The position of a pixel in key matrices is performed with the use of just (2 × 3 × 356) of extra space and a pre-computation time off (256). The time optimization of the algorithm reflects when encrypting images for large sizes. Both key matrices are encrypted using ElGamal encryption before sending it to the receiver. The key maps can be easily generated on the receiver end once the symmetric keys are shared and derived.
**Algorithm 4:** ElGamal with Double Playfair Cipher  **Input**: The image of size 256 × 256 to be encrypted or decrypted.  The key matrices corresponding to Double Playfair Cipher.  The ElGamal cryptosystem parameters.  **Output**: The corresponding cipher image or original image of size 256 × 256.  1.  Key Generation for Double Playfair Cipher    (a) A modified key space of 2 matrices, each of size 16 × 16 is used instead of standard       5 × 5 key matrices used in Double Playfair cipher.    (d) Create 2 key maps, 1 corresponding to each of the keys. The key maps have pixel       intensities as keys and their location in the respective key matrix as the       corresponding value (represented by a tuple of row and column).  2.  Key Generation for ElGamal Cryptosystem    (a) Receiver chooses a prime number *p* and computes its generator g≡gd(modp)    (b) Publish the public key, prime number *p*, and generator *g*.    (c) Sender chooses a random integer *i* in the range: {2,p−2}.    (d) Sender computes the temporary key or ephemeral key as: $KE=g_{i}\left(mod\;p\right)$    (e) The masking key is computed by sender as: $KE\equiv K_{pubi}\left(mod\;p\right)$.  3.  Encryption process    (a) Read the image to be encrypted and separate it to different channels as R, G, B.       Pair up pixels in 2 for each of the channels separately.    (b) To apply Vertical Double Playfair Cipher find the location of first value from pixel       pair from the first key map and the location of second pixel value from the second       key map. Then if the pixels are in same column keep them unchanged, else find the       pixel intensities at the opposite corners of the rectangle formed by the pair of input       pixels considered. Place them in the same order as they correspond to the order of       pixel values in the input pair.    (c) Substitute the cipher pixel values to create the cipher image of size identical to that       of the input image.    (d) Encrypt each of the key matrices using the masking key from ElGamal Encryption as:       $c_{ij}\equiv p_{ij}\times KM\left(mod\;p\right)$       where $p_{ij}$ denotes the corresponding pixel intensity in a cell of a key matrix.    (e) Send the encrypted image and encrypted key matrices along with ElGamal public key       (ephemeral key) of sender to the receiver.  4.  Decryption process    (a) The masking key will be computed by receiver as: KM≡KE(modp).    (b) The key matrices for Double Playfair Cipher are decrypted as:       $p_{ij}=c_{ij}\times \left(KM-1\right)\left(mod\;p\right)$.    (c) Read the image to be decrypted and separate it to different channels as R, G, B.       Pair up pixels in 2 for each of the channels separately.    (d) To apply Vertical Double Playfair Cipher find the location of first value from pixel       pair from the first key map and the location of second pixel value from the second       key map. Then if the pixels are in same column, keep them unchanged.       Otherwise, find the pixel intensities at the opposite corners of the rectangle       formed by the pair of input pixels considered. Place them in the same order as they       correspond to the order of pixel values in the input pair.    (e) Substitute the decrypted pixel values to create the original image.

## 4. Image Encryption and Decryption Implementation

**Figure 2:**
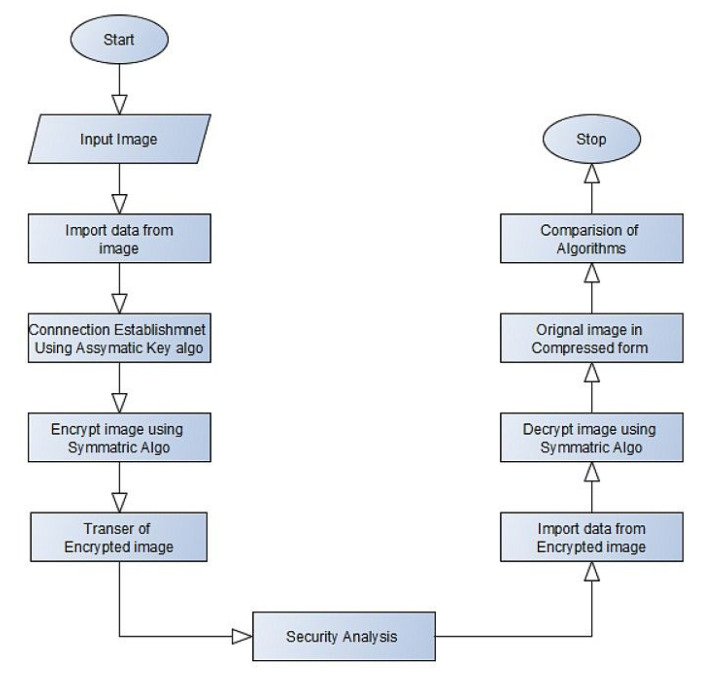
Flow chart for image encryption and decryption.

**Figure 3:**
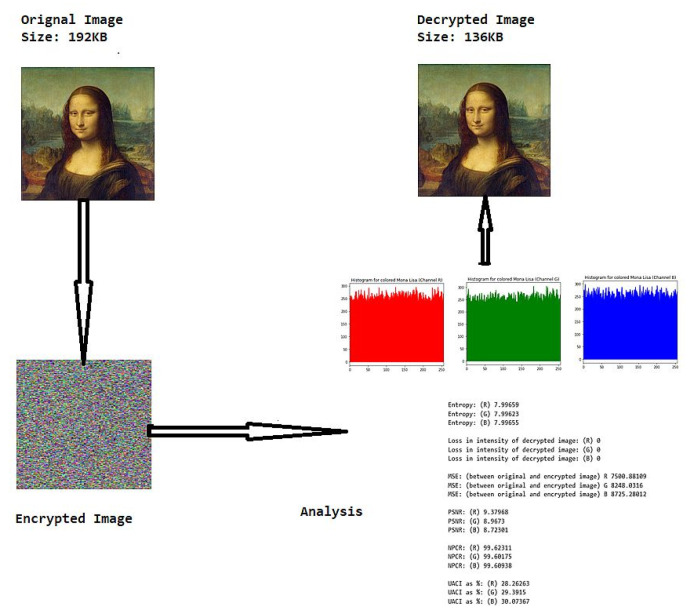
Parameter analysis of encryption and decryption.

The flow diagram for the proposed hybrid encryption and decryption algorithm is shown in Figure 2 and Figure 3. A connection is established using the key generated by the Elliptical Curve Cryptography (ECC) asymmetric key algorithm. The image encryption is by the symmetric hybrid algorithm of Elliptic Curve Cryptography (ECC) with Hill Cipher, ECC with Advanced Encryption Standard (AES), and ElGamal with Double Playfair Cipher. The encrypted image is securely sent to the sender. The image is decrypted by the receiver using this symmetric hybrid algorithm, and the original image retrieved will be compressed.

## 5. Performance Analysis of Proposed Hybrid Algorithms

In this section, we discuss within detail the proposed hybrid algorithms using various symmetrical and asymmetrical techniques for image encryption. To compare all the different methods that have been incorporated further, we have established such guidelines, both general and specific to an algorithm. The rules to be followed when choosing certain criteria to perform encryption and decryption correctly. The complete set of rules helps us to carry out a comparative analysis of each algorithm and also to evaluate the work done using the same parameters set for each process.

### 5.1. General Constraints

The general constraints to be followed for image selection are

- The size of the image to be encrypted is 256×256 pixels. The size of the encrypted image is the same as the input image except for the AES with ECC algorithm where the size of the encrypted image is slightly larger. All decryption algorithms produce a decrypted image of the same size as the original image.
- To hold both Grayscale and RGB images as the user’s choice, we deploy an algorithm that both encrypts and decrypts all 3 channels (RGB) of the picture separately.

### 5.2. Parameters for Comparison

The comparative analysis performed on the basis of the following parameters. Test samples shown for ECC with Hill Cipher, ECC with AES and ElGamal with Double Playfair Cipher.

- Encryption Time: Encryption is the method used to convert information to a secret code that masks the true meaning of the information. The time it takes to encrypt the image is the time to encrypt it.
- Decryption Time: Decryption is generally the reverse encryption process. It is the method of decoding data that has been encrypted in a hidden format. The time it takes to get the original image back is the time to decrypt it.
- Metric Values for Entropy: 8 expected value for good Encryption process. Entropy is the randomness of the pixel intensities in the encrypted image. An entropy value close to 8 is a good encryption algorithm for an 8-bit image. Values obtained for Eggs (Grayscale), Eggs (Coloured), Mona Lisa (Grayscale) and Mona Lisa (Coloured) are closure to 8.
- Metric Values for PSNR (dB): 10 dB expected value for reconstructed image. The PSNR block calculates the peak signal-to-noise ratio between two images in decibels. This ratio is used to measure the quality between the original and the compressed image. The higher the PSNR, the higher the quality of the compressed or reconstructed image. Values obtained for Eggs (Grayscale), Eggs (Coloured), Mona Lisa (Grayscale) and Mona Lisa (Coloured) are between 8 and 9.5.
- Metric Values for NPCR (%): 100% expected value for varying number of pixels from the input image in the encrypted image. NPCR is the change in the number of pixels of the cipher image when only one pixel of the plain image is changed. It is an indicative measure of the number of pixels that vary from the input image in the encrypted image and is expressed as a percentage. Values obtained for Eggs (Grayscale), Eggs (Coloured), Mona Lisa (Grayscale) and Mona Lisa (Coloured) are closure to 100%.
- Metric Values for UACI (%): 30% expected value for varying number of pixels from the input image in the encrypted image. UACI measure indicates the security of the algorithm against differential attacks, such as a plaintext attack, a cipher-only attack, or a known plaintext attack. Higher value indicates that it is safer against such attacks. Values obtained for Eggs (Grayscale), Eggs (Coloured), Mona Lisa (Grayscale) and Mona Lisa (Coloured) are between 26% to 30.5%.
- Squared Error in Decrypted image: The discrepancy between the decrypted image pixels and the original one. It should be closer to zero for a good algorithm.

## 6. Evaluation and Result Analysis

The various test cases help us visualize the input image, the encrypted image and the decrypted image for each of the proposed hybrid algorithms. The test cases performed for input samples of grayscale and coloured images. The evaluation carried out on metric measurements, visualized maps and histogram analysis.

### 6.1. Sample Input and Output

The reference input and output images used for study are Mona Lisa (Grayscale 256 × 256 Pixels), Mona Lisa (Coloured 256 × 256 Pixels), Eggs (Grayscale 256 × 256 Pixels), Eggs (Coloured 256 × 256 Pixels). The sample input and output with proposed encryption and decryption is shown in Table 1.

### 6.2. Metric Measurements

The tuple in the table indicates the value of the metrics in the order of (R, G, and B).

#### 6.2.1. Eggs (Grayscale 256 × 256 Pixels) Image

The metric measurements such as Entropy, PSNR, NPCR, UACI and Mean Squared Error (MSE) are shown in Table 2 for Eggs (Grayscale) image.

#### 6.2.2. Eggs (coloured 256×256 Pixels) Image

The Entropy, PSNR, NPCR, UACI and Mean Squared Error (MSE) metrics for Eggs (coloured) image are shown in Table 3.

#### 6.2.3. Mona Lisa (Grayscale 256×256 Pixels) Image

The Metric measurements such as Entropy, PSNR, NPCR, UACI and Mean Squared Error (MSE) are shown in Table 4 for Mona Lisa (Grayscale) image.

#### 6.2.4. Mona Lisa (Coloured 256×256 Pixels) Image

The Entropy, PSNR, NPCR, UACI and Mean Squared Error (MSE) metrics for Mona Lisa (coloured) image are shown in Table 5.

### 6.3. Histogram Analysis of the Encrypted Images

Table 6 shows histogram of red, green and blue component of gray scale and coloured image (Mona Lisa). It is clearly visible that histogram of cipher image is fairly uniform and it does not leak any amount of information about the plain image

Table 7 shows histogram of red, green and blue component of gray scale and coloured image (Egg). It is clearly visible that histogram of cipher image is fairly uniform and it does not leak any amount of information about the plain image.

## 7. Discussion and Comparative Analysis

### 7.1. Discussion

The different performance metrics measured in our work evaluate the security of the encryption provided by the hybrid algorithm to an input image. The entropy value obtained for Encrypted Image (ECC with Hill Cipher), Encrypted Image (ECC with AES), and Encrypted Image (ElGamal with Double Playfair cipher) signifies the randomness of pixel intensities in the encrypted image. Test case performed for a set of gray and coloured images. An entropy value obtained closure to 8 for the sample 8-bit image implies a strong encryption algorithm.

Similarly, in the encryption process, a lower value obtained from PSNR implies greater randomness. Using the entropy and PSNR measurement value, ECC with AES and ECC with Hill Cipher introduce significantly high randomness in image encryption compared to ElGamal with Double Playfair cipher algorithms

NPCR is also a representative measure of the number of pixels that vary from the input image in the encrypted image and are expressed as a percentage value. While the NPCR value for the ECC algorithm with a mapping table is 100%, the lower entropy implies that the probability distribution of the different pixel intensities is not uniform. Test samples show that the NPCR values for ECC with Hill Cipher and ECC with AES are very close to 100%.

The UACI measure indicates the security of the algorithm against differential attacks, such as a plaintext attack chosen, a ciphertext-only attack, or a known-plaintext attack. A higher UACI value for ECC with AES and ECC with Hill Cipher suggests that algorithms are more secure against such attacks. While we can also infer that the UACI value for ElGamal with the Double Playfair algorithm is very close to the previous algorithms. However, we can also deploy ElGamal with Double Playfair Cipher for applications that require ease of implementation, speed, and large image sizes. However, the algorithm fails to combat brute-force attacks requiring the knowledge of a large number of ciphertexts. This can be minimized by deploying a new pair of symmetric keys for each set of communications to protect the ElGamal cryptosystem. While communication overhead increases slightly, the algorithm can be found to be very useful for applications involving less frequency of communication between parties.

The NPCR and UACI are designed to test the number of changing pixels and the number of averaged changed intensity between ciphertext images. The upper-bound of the NPCR score is 100%, and thus it is believed that the NPCR score of a secure cipher should be very close to this upper-bound. Experimental results show the estimated expectations and variance of NPCR and UACI are very close to the expected values. NPCR or UACI, are random variables dependent on parameters such as the image size and the format of the image rather than static values. This shows more resistive to the cipher attack.

### 7.2. Comparative Analysis

The image encryption and decryption are constructed with our proposed hybrid algorithms and referred in Table 8.

## 8. Conclusions

Digital images are sensitive images that must be secured against intruders through network channel transmission. Different imaging techniques are used to encrypt images using symmetrical asymmetric and encryption algorithms. Many of the image algorithms support only either symmetrical encryption and decryption or asymmetric encryption and decryption. Thus we have researched three different hybrid methods for the implementation of image encryption such as Hill Cipher ECC, Advanced Encryption Standard ECC, and Double Playfair Cipher ElGamal. Test cases performed with a set of grayscale and coloured images and performance metrics are measured. When calculating all the parameters such as Encryption Time, Decryption Time, Entropy, Squared Error in Decrypted Image, PSNR, NPCR, UACI, we can infer which algorithm is better suited to the needs of the user. The result shows effectiveness with lower values of encryption and decryption time. The proposed hybrid algorithm Entropy Value obtained has closure to 8 which is better than existing algorithms. We obtain the lesser value for Squared Error in the Decrypted image which proved better than other algorithms. However, the value of the PSNR is higher by the metric scales and the higher the value of NPCR the better the algorithm is, and higher value of UACI indicates it is more secured from attacks. Based on the time taken for encryption and decryption, we can recognize that ECC with AES is computationally intensive and not feasible for applications involving the protection of large image databases. We have performed metric measures for two gray scale and two colour images of 256×256 pixels Eggs and Mona Lisa. The Entropy, PSNR, UACI, and NPCR metric measures are effective and have closure to their expected values. So we will extend this as future work with various images of varying sizes in pixels. ECC with AES appears to be good for remote or private communications with smaller image sizes due to the following reasons: (1) Additional bandwidth is not required for AES, (2) key length of ECC occupies less storage space, and (3) ECC requires shorter bandwidth for decryption. To conclude, the ECC and Hill Cipher turn out to be a good overall alternative for image encryption algorithms.

## Figures and Tables

**Table 1:** Sample input and output for proposed hybrid algorithms.

Image Name	Original Image	Encrypted Image (ECC with Hill Cipher)	Encrypted Image (ECC with AES)	Encrypted Image (ElGamal with Double Playfair) Cipher	Encrypted Image (Decrypted Image)
Mona Lisa (Grayscale)	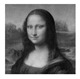	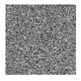	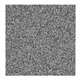	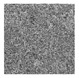	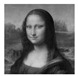
Mona Lisa (Coloured)	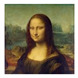	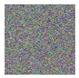	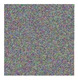	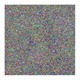	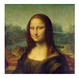
Egg (Grayscale)	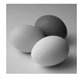	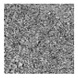	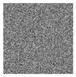	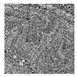	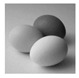
Egg (Coloured)	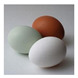	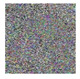	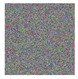	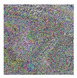	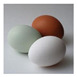

**Table 2:** Metric measures for Eggs (grayscale).

Evaluation Metrics	ECC with Hill Cipher	ECC with AES	ElGamal with Double Playfair Cipher
Encryption Time (seconds)	0.33418	2.82401	0.18775
Decryption Time (seconds)	0.36932	2.75127	0.17856
Entropy	7.9434	7.99653	7.78708
Mean Squared Error (MSE) in decrypted image	0.0000	0.0000	0.0000
PSNR (dB)	9.45457	9.129	9.36168
NPCR (%)	86.91254	99.60632	94.81812
UACI (%)	26.06152	28.90828	27.91177

**Table 3:** Metric measures for Mona Lisa (coloured).

Evaluation Metrics	ECC with Hill Cipher	ECC with AES	ElGamal with Double Playfair Cipher
Encryption Time (seconds)	0.32915	2.52632	0.13013
Decryption Time (seconds)	00.35193	2.50829	0.1486
Entropy	(7.99701, 7.9971, 7.98925)	(7.99667, 7.99659, 7.99672)	(7.95568, 7.96154, 7.8657)
Mean Squared Error (MSE) in decrypted image	(0, 0, 0)	(0, 0, 0)	(0, 0, 0)
PSNR (dB)	(8.80367, 8.45589, 7.91148)	(8.86691, 8.54116, 7.9478)	(9.19181, 8.8655,8.27107)
NPCR (%)	(99.48273, 99.38507, 99.47968)	(99.63379, 99.64142, 99.6109)	(93.57605, 93.23425, 93.7561)
UACI (%)	(29.85083, 31.02715, 32.87405)	(29.60341, 30.65091, 32.69575)	(27.70115, 28.5317, 30.3973)

**Table 4:** Metric measures for Mona Lisa (gray).

Evaluation Metrics	ECC with Hill Cipher	ECC with AES	ElGamal with Double Playfair Cipher
Encryption Time (seconds)	0.44584	2.84150	0.24519
Decryption Time (seconds)	0.35107	2.7866	0.20224
Entropy	7.99442	7.99671	7.92129
Mean Squared Error (MSE) in decrypted image	0.0000	0.0000	0.0000
PSNR (dB)	8.5959	8.62078	9.12066
NPCR (%)	97.41669	99.58191	94.13757
UACI (%)	30.26131	30.37332	27.97823

**Table 5:** Metric measures for Eggs (grayscale).

Evaluation	ECC with	ECC with	ElGamal with Double
Metrics	Hill Cipher	AES	Playfair Cipher
Encryption Time (s)	0.32507	2.53642	0.13366
Decryption Time (s)	0.35904	2.52728	0.16954
Entropy	(7.98534, 7.98371, 7.98925)	(7.99652, 7.99585, 7.99638)	(7.85019, 7.87646, 7.90649)
Mean Squared Error (MSE) in decrypted image	(0, 0, 0)	(0, 0, 0)	(0, 0, 0)
PSNR (dB)	(9.41053, 8.95845, 8.74391)	(9.39128, 8.98602, 8.7218)	(9.86406, 9.45456, 9.069)
NPCR (%)	(96.67358, 96.81854, 96.99249)	(99.646, 99.63684, 99.62463)	(94.05823, 94.08875, 94.1803)
UACI (%)	(27.87552, 29.14918, 29.73587)	(28.15297, 29.32043, 30.11092)	(26.05094, 27.09534, 28.17803)

**Table 6:** Histogram analysis for encrypted image (Mona Lisa) using hybrid algorithms.

Image	Encrypted Image (ECC with Hill Cipher)	Encrypted Image (ECC with AES)	Encrypted Image (ElGamal with Double Playfair Cipher)
Mona Lisa (Grayscale)	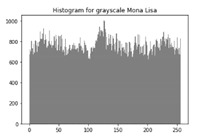	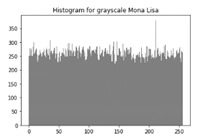	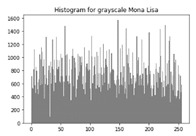
Mona Lisa (Coloured)	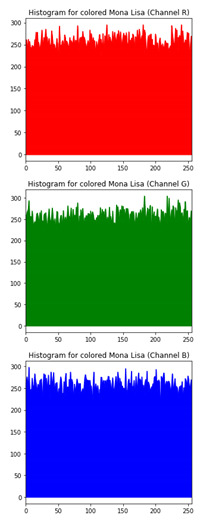	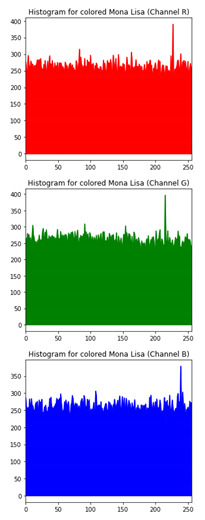	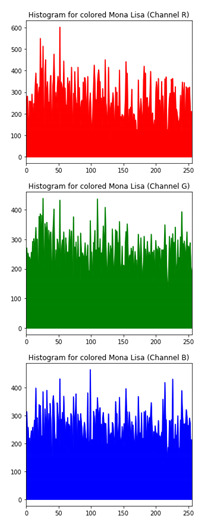

**Table 7:** Histogram analysis for encrypted image (Egg) using hybrid algorithms.

Image	Encrypted Image (ECC with Hill Cipher)	Encrypted Image (ECC with AES)	Encrypted Image (ElGamal with Double Playfair Cipher)
Egg (Grayscale)	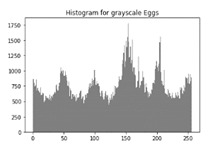	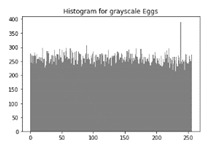	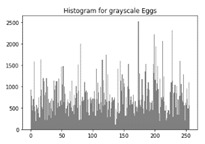
Egg (Coloured)	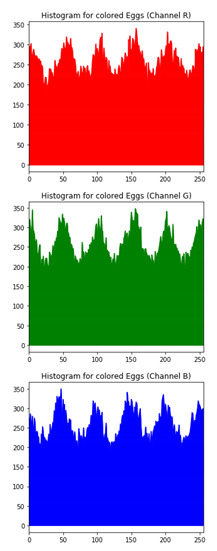	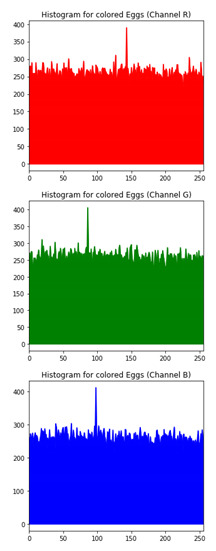	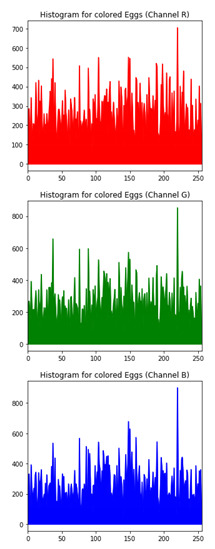

**Table 8:** Comparative analysis of proposed hybrid algorithms.

S. No.	Image Encryption and Decryption Algorithms	Proposed Hybrid Algorithms
1	Encryption and Decryption algorithms proposed either for only symmetric or Asymmetric Encryption and Decryption only.	The proposed symmetric and Asymmetric hybrid algorithms are ECC with Hill Cipher, ECC with AES and ElGamal with Double Playfair cipher.
2	Symmetric Key provides faster processing, less protection for message transfer.	Proposed algorithm solves the problem of computation and high protection.
3	Several techniques adopted without compression of the images required more space.	The techniques proposed here compress the image, which is the less space needed to provide the same information.
4	Requires high bandwidth without compression.	We have proposed with compression that results in a low bandwidth, less storage space and less computation time.
5	Most researchers use only some measure for the security analysis.	Different metric measures such as Entropy, PSNR, NPCR, and UACI in decrypted image are taken into account.
